# Use of Voice-Based Conversational Artificial Intelligence for Basal Insulin Prescription Management Among Patients With Type 2 Diabetes: A Randomized Clinical Trial

**DOI:** 10.1001/jamanetworkopen.2023.40232

**Published:** 2023-12-01

**Authors:** Ashwin Nayak, Sharif Vakili, Kristen Nayak, Margaret Nikolov, Michelle Chiu, Philip Sosseinheimer, Sarah Talamantes, Stefano Testa, Srikanth Palanisamy, Vinay Giri, Kevin Schulman

**Affiliations:** 1 Division of Hospital Medicine, Stanford University School of Medicine, Stanford, California; 2 Division of Primary Care and Population Health, Stanford University School of Medicine, Stanford, California; 3 Clinical Excellence Research Center, Stanford University School of Medicine, Stanford, California; 4 Department of Medicine, Stanford University, Stanford, California; 5 Graduate School of Business, Stanford University, Stanford, California

## Abstract

**Importance:**

Optimizing insulin therapy for patients with type 2 diabetes can be challenging given the need for frequent dose adjustments. Most patients receive suboptimal doses and do not achieve glycemic control.

**Objective:**

To examine whether a voice-based conversational artificial intelligence (AI) application can help patients with type 2 diabetes titrate basal insulin at home to achieve rapid glycemic control.

**Design, Setting, and Participants:**

In this randomized clinical trial conducted at 4 primary care clinics at an academic medical center from March 1, 2021, to December 31, 2022, 32 adults with type 2 diabetes requiring initiation or adjustment of once-daily basal insulin were followed up for 8 weeks. Statistical analysis was performed from January to February 2023.

**Interventions:**

Participants were randomized in a 1:1 ratio to receive basal insulin management with a voice-based conversational AI application or standard of care.

**Main Outcomes and Measures:**

Primary outcomes were time to optimal insulin dose (number of days needed to achieve glycemic control), insulin adherence, and change in composite survey scores measuring diabetes-related emotional distress and attitudes toward health technology and medication adherence. Secondary outcomes were glycemic control and glycemic improvement. Analysis was performed on an intent-to-treat basis.

**Results:**

The study population included 32 patients (mean [SD] age, 55.1 [12.7] years; 19 women [59.4%]). Participants in the voice-based conversational AI group more quickly achieved optimal insulin dosing compared with the standard of care group (median, 15 days [IQR, 6-27 days] vs >56 days [IQR, >29.5 to >56 days]; a significant difference in time-to-event curves; *P* = .006) and had better insulin adherence (mean [SD], 82.9% [20.6%] vs 50.2% [43.0%]; difference, 32.7% [95% CI, 8.0%-57.4%]; *P* = .01). Participants in the voice-based conversational AI group were also more likely than those in the standard of care group to achieve glycemic control (13 of 16 [81.3%; 95% CI, 53.7%-95.0%] vs 4 of 16 [25.0%; 95% CI, 8.3%-52.6%]; difference, 56.3% [95% CI, 21.4%-91.1%]; *P* = .005) and glycemic improvement, as measured by change in mean (SD) fasting blood glucose level (−45.9 [45.9] mg/dL [95% CI, −70.4 to −21.5 mg/dL] vs 23.0 [54.7] mg/dL [95% CI, −8.6 to 54.6 mg/dL]; difference, −68.9 mg/dL [95% CI, −107.1 to −30.7 mg/dL]; *P* = .001). There was a significant difference between the voice-based conversational AI group and the standard of care group in change in composite survey scores measuring diabetes-related emotional distress (−1.9 points vs 1.7 points; difference, −3.6 points [95% CI, −6.8 to −0.4 points]; *P* = .03).

**Conclusions and Relevance:**

In this randomized clinical trial of a voice-based conversational AI application that provided autonomous basal insulin management for adults with type 2 diabetes, participants in the AI group had significantly improved time to optimal insulin dose, insulin adherence, glycemic control, and diabetes-related emotional distress compared with those in the standard of care group. These findings suggest that voice-based digital health solutions can be useful for medication titration.

**Trial Registration:**

ClinicalTrials.gov Identifier: NCT05081011

## Key Points

**Question:**

Can a voice-based conversational artificial intelligence (AI) application help patients with type 2 diabetes titrate basal insulin at home to achieve rapid glycemic control?

**Findings:**

In this randomized clinical trial that included 32 adults with type 2 diabetes requiring initiation or adjustment of basal insulin, participants who used a voice-based conversational AI application had a significantly improved time to optimal insulin dose (median, 15 days vs >56 days) and insulin adherence (83% vs 50%) compared with participants receiving standard of care.

**Meaning:**

Patient-facing, voice-based conversational AI applications can help patients with type 2 diabetes quickly achieve basal insulin dose optimization.

## Teaser

This randomized clinical trial examines whether a voice-based conversational artificial intelligence (AI) application can help patients with type 2 diabetes titrate basal insulin at home to achieve rapid glycemic control.

## Introduction

Nearly one-fourth of the 33 million US adults with type 2 diabetes have poor glycemic control with a hemoglobin A_1c_ (HbA_1c_) level above 8%.^1,2,3^ Insulin therapy is essential for individuals with poorly controlled diabetes, but effective use requires frequent dose titrations,^4^ which can be challenging to achieve in practice because titrations typically occur at outpatient clinic visits every 3 to 6 months.^5^ In addition, many clinicians fail to escalate insulin therapy when indicated due to therapeutic inertia, lack of time, and competing demands in appointments.^6,7,8,9,10^ As a result, most patients treated with insulin receive suboptimal doses and do not achieve glycemic control.^11,12^

Self-titration of insulin by patients is a potential solution to overcome these barriers. Several studies have shown self-titration to be safe and effective.^13,14,15,16,17,18,19^ However, this approach requires ongoing education and monitoring to ensure that patients appropriately follow dosing instructions.^20,21^ To achieve this, some health systems use care management teams with nurses and pharmacists to assist patients with protocolized dose adjustments.^22^ Digital health tools, such as mobile apps and remote patient monitoring devices, can offer a more scalable approach. Historically, most of these tools have focused on diabetes education and medication reminders, but there is an increasing number of apps and devices that provide real-time decision support for insulin self-titration.^23,24,25,26,27^ Work is ongoing to develop and validate effective digital health tools in this space.

In this study, we developed a voice-based conversational artificial intelligence (VBAI) application to help patients with type 2 diabetes manage basal insulin titration at home. We chose a voice-based interface over the more commonly used smartphone interface because of its potential to improve access, usability, and convenience, especially for older patients with diabetes.^28,29,30,31^ We evaluated the effectiveness of the VBAI in medication adherence, glycemic control, and time to optimal insulin dose compared with standard of care. To our knowledge, this study marks the first time a VBAI has been used for medication titration.

## Methods

### Trial Design

The Managing Insulin with Voice AI (MIVA) trial was a remote (decentralized), randomized, open-label, parallel-group clinical trial investigating a novel VBAI application for basal insulin titration compared with standard of care. The trial was conducted at 4 primary care clinics at Stanford University from March 1, 2021, to December 31, 2022. The protocol was approved by the Stanford University institutional review board (trial protocol in Supplement 1). All participants completed an online informed consent form. This study followed the Consolidated Standards of Reporting Trials (CONSORT) reporting guideline.

### Recruitment, Enrollment, and Randomization

We recruited English-speaking adults with type 2 diabetes who required initiation or adjustment of once-daily basal insulin. Exclusion criteria were the use of insulin pumps or the inability to independently carry out the intervention (ie, technical barriers in the home).

Participants were randomly assigned in a 1:1 ratio to receive basal insulin management with VBAI or standard of care. The randomization schedule was computer-generated and stratified by age and self-reported gender with a permuted-block design (random block sizes of 2 and 4). Participants were followed up for 8 weeks.

### Interventions

All participants completed a demographics intake form recording age, gender, race, and ethnicity. They also filled out 3 surveys: the 5-item Problem Areas in Diabetes Scale (PAID-5), a survey on diabetes-related emotional distress; a 5-question survey on attitudes toward medication adherence; and a 2-question survey on attitudes toward health technology (eAppendices 1-3 in Supplement 2).^32^ All participants repeated these 3 surveys at 8 weeks. Survey items were scored using a 5-point Likert scale from 0 to 4 (PAID-5 survey: 0 indicated not a problem and 4 indicated serious problem; attitudes toward medication adherence survey: 0 indicated strongly agree and 4 indicated strongly disagree; and attitudes toward health technology survey: 0 indicated strongly agree and 4 indicated strongly disagree). Surveys were collected using REDCap, a secure web-based platform designed for research studies.^33,34^

### Voice-Based Conversational AI

We developed custom voice AI software for this trial powered by Alexa, a Health Insurance Portability and Accountability Act–compliant conversational AI platform by Amazon.^35^ All software development was conducted independently by the research team without funding; Amazon was not involved in this study. The VBAI was deployed on an Amazon smart speaker. All participant interactions occurred through voice commands and short conversations. The function of the VBAI was to assist the participant with at-home titration of basal insulin. The VBAI was rules based and deterministic, based on titration algorithms by the American Association of Clinical Endocrinologists and the American College of Endocrinology, and included emergency protocols to handle hypoglycemia and hyperglycemia (eMethods in Supplement 2).^4^ Software development and beta testing were conducted from June 1, 2020, to February 28, 2021.

Participants received an Amazon smart speaker loaded with the custom VBAI. Prior to activation, the participant’s primary diabetes clinician (primary care professional, endocrinologist, or clinical pharmacist) selected an insulin titration protocol via a custom web portal (eFigure 1 in Supplement 2). Protocol parameters included a starting insulin dose, a goal fasting blood glucose (FBG) level range, and insulin titration instructions. Once the protocol was approved, participants were instructed to check in with the VBAI daily, using the phrase “Alexa, check in with clinical trial.” This phrase triggered a conversation in which the participant would report clinical data, such as recent insulin use and FBG values. At the end of the conversation, the VBAI provided updated insulin dosing instructions based on these data. All data were available in real-time on our portal for clinicians and the research team (eFigures 2-4 in Supplement 2).

### Standard of Care

Participants randomized to the standard of care group had basal insulin titrated by their clinician per usual care. They received an online blood glucose and insulin log, which they were instructed to fill out daily for the duration of the trial (eAppendix 4 in Supplement 2). They also received an Amazon smart speaker, which was set up with daily reminders to complete their log; they did not have access to our VBAI.

### Outcomes

The key primary outcome was time to optimal insulin dose, measured as the number of days between the study start date and the date that the goal 3-day mean FBG level was achieved. Other primary outcomes were mean insulin adherence based on logged data and change in the composite scores of the 3 surveys measuring attitudes toward diabetes, health technology, and medication adherence. Secondary outcomes were glycemic control and glycemic improvement. Glycemic control was measured as the proportion of participants who had achieved the goal FBG level by 8 weeks. Glycemic improvement was measured as the change in 3-day mean FBG values from baseline to 8 weeks.

### Sample Size

Based on a similar study, sample size was determined to be 32 participants.^26^ With this sample size, the statistical power was 80%, with a 2-sided α of .05, to detect a treatment difference of 84% for the VBAI group vs 40% for the standard of care group for the proportion of participants who achieved glycemic control.

### Statistical Analysis

Analysis was performed on an intent-to-treat basis. The primary outcome of the time to optimal insulin dose was assessed using the log-rank test to compare time to event for the VBAI group vs standard of care. Standard methods for mean values and proportions were used to construct 95% CIs and to conduct tests. Specifically, the Welch 2-sample method was used to test for differences in insulin adherence, change in composite survey scores (baseline to 8 weeks), and change in 3-day mean FBG level (baseline to 8 weeks) between VBAI and standard of care groups. The 2-sample test for equality of proportions with the Yates continuity correction was used to assess differences in glycemic control.

We reviewed participants’ medical records to supplement any missing data for our outcomes related to time to optimal insulin dose, mean glycemic control, and glycemic improvement. If FBG values were incomplete in the final 3 days of the trial, the last 3 available FBG values were used.

Analyses were performed from January to February 2023 using R statistical software, version 4.2.1 (R Project for Statistical Computing). Statistical significance was defined as 2-sided *P* < .05.

## Results

### Study Participants

**Figure 1.**
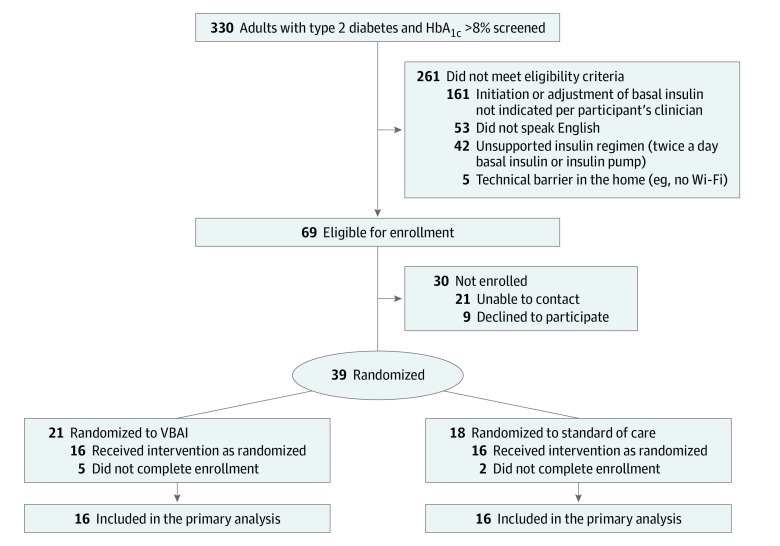
Patient Flow Diagram HbA_1c_ indicates hemoglobin A_1c_; VBAI, voice-based conversational artificial intelligence.

Between March 1, 2021, and October 31, 2022, 330 individuals were screened for eligibility, 39 participants were randomized, and 32 participants completed the enrollment process (Figure 1). All 32 participants were included in the analysis of the primary outcome of time to optimal insulin dose. The mean (SD) age was 55.1 (12.7) years (range, 30-74 years); 19 participants were women (59.4%); and 2 (6.3%) were African American, 1 (3.1%) was American Indian, 8 (25%) were Asian, 8 (25%) were Hispanic, and 16 (50%) were White (Table 1). The mean (SD) HbA_1c_ level was 9.6% (1.5%) (to convert to proportion of total hemoglobin, multiply by 0.01). Baseline characteristics are shown in Table 1.

**Table 1.** Baseline Characteristics of Enrolled Participants

Characteristic	Total (N = 32)	Standard of care (n = 16)	VBAI (n = 16)
Age, mean (SD), y	55.1 (12.7)	56.2 (13.5)	54.1 (12.3)
Gender, No. (%)			
Female	19 (59.4)	10 (62.5)	9 (56.3)
Male	13 (40.6)	6 (37.5)	7 (43.8)
Race and ethnicity, No. (%)^a^			
White	16 (50.0)	8 (50.0)	8 (50.0)
Asian	8 (25.0)	5 (31.3)	3 (18.8)
American Indian or Alaska Native	1 (3.1)	0	1 (6.3)
Black or African American	2 (6.3)	1 (6.3)	1 (6.3)
>1 Race	3 (9.4)	1 (6.3)	2 (12.5)
Unknown or not reported	2 (6.3)	1 (6.3)	1 (6.3)
Hispanic ethnicity, No. (%)^a^	8 (25.0)	5 (31.3)	3 (18.8)
HbA_1c_, mean (SD), %	9.6 (1.5)	9.3 (1.4)	9.9 (1.6)
PAID-5 score, mean (SD)^b^	7.5 (4.3)	6.5 (4.9)	8.4 (3.4)
Attitudes toward medication adherence score, mean (SD)^b^	13.8 (4.1)	13.6 (3.8)	14.0 (4.4)
Attitudes toward health technology score, mean (SD)^b^	6.9 (1.7)	6.6 (1.9)	7.1 (1.5)

Abbreviations: HbA_1c_, hemoglobin A_1c_; PAID-5, Problem Areas in Diabetes Questionnaire–5 item; VBAI, voice-based conversational artificial intelligence.

SI conversion factor: To convert HbA_1c_ to proportion of total hemoglobin, multiply by 0.01.

^a^ Race and ethnicity were self-reported by the participant.

^b^ Survey items were scored from 0 to 4, and composite scores were calculated as the sum across all items.

### Outcomes

**Figure 2.**
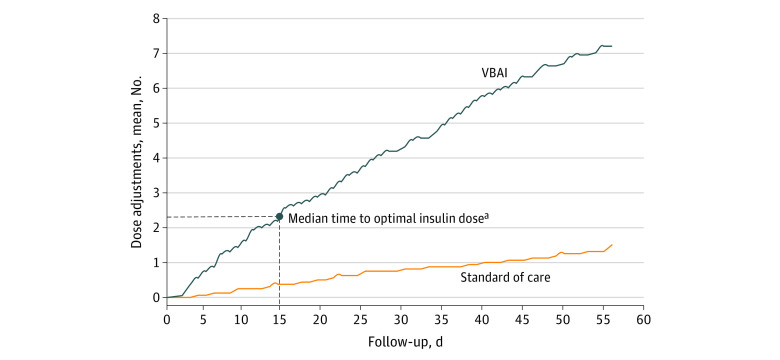
Number of Insulin Dose Adjustments per Participant Over Time VBAI indicates voice-based conversational artificial intelligence. ^a^The median time to optimal insulin dose was 15 days in the VBAI group. Less than 50% of participants achieved optimal insulin dosing in the standard of care group.

The time to optimal insulin dose was different for participants in the VBAI group compared with the standard of care group; the median time to optimal insulin dose was 15 days (IQR, 6-27 days) for the VBAI group and exceeded 56 days (IQR, >29.5 to >56 days; significant difference in time-to-event curves; *P* = .006) for the standard of care group, where fewer than half of participants achieved optimal insulin dosing at 8 weeks. As shown in Table 2, participants in the VBAI group achieved a mean (SD) insulin adherence of 82.9% (20.6%) compared with 50.2% (43.0%) in the standard of care group (difference, 32.7% [95% CI, 8.0%-57.4%]; *P* = .01). Participants in the VBAI group had a mean (SD) of 7.3 (4.2) automated insulin dose adjustments compared with 1.6 (3.2) dose adjustments in the standard of care group (Figure 2).

**Table 2.** Primary and Secondary Outcomes

Outcome	VBAI	Standard of care	Difference: VBAI vs standard of care (95% CI)	*P* value
Primary outcomes				
Insulin adherence, mean (SD), %	82.9 (20.6)	50.2 (43.0)	32.7 (8.0 to 57.4)	.01
Change in PAID-5 score, mean (SD)^a^	−1.9 (4.2)	1.7 (4.4)	−3.6 (−6.8 to −0.4)	.03
Change in attitudes toward health technology score, mean (SD)^a^	0.3 (1.4)	−1.1 (2.3)	1.4 (−0.03 to 2.8)	.06
Change in attitudes toward medication adherence score, mean (SD)^a^	0.8 (4.3)	−0.1 (2.6)	1.0 (−1.6 to 3.5)	.46
Secondary outcomes				
Proportion who achieved glycemic control	0.81	0.25	0.56 (0.21 to 0.91)	.005
Change in FBG level, mean (SD), mg/dL	−45.9 (45.9)	23.0 (54.7)	−68.9 (−107.1 to −30.7)	.001

Abbreviations: FBG, fasting blood glucose; PAID-5, 5-item Problem Areas in Diabetes Questionnaire; VBAI, voice-based conversational artificial intelligence.

SI conversion factor: To convert glucose to millimoles per liter, multiply by 0.0555.

^a^ Survey items were scored from 0 to 4, and composite scores were calculated as the sum across all items. Change was calculated as the composite score at 8 weeks minus the composite score at baseline.

Of the 32 participants, 31 completed the 3 study surveys at 8 weeks. PAID-5 survey scores decreased by a mean of 1.9 points (95% CI, −4.1 to 0.4 points) in the VBAI group, but increased by a mean of 1.7 points (95% CI, −0.7 to 4.2 points) in the standard of care group, for a mean difference of −3.6 points (95% CI, −6.8 to −0.4 points; *P* = .03). Attitudes toward health technology survey scores increased by a mean of 0.3 points (95% CI, −0.5 to 1.0 points) in the VBAI group but decreased by a mean of 1.1 points (95% CI, −2.4 to 0.1 points) in the standard of care group, for a mean difference of 1.4 points (95% CI, −0.03 to 2.8 points; *P* = .06). Attitudes toward medication adherence survey scores increased by a mean of 0.8 points (95% CI, −1.5 to 3.1 points) in the VBAI group and decreased by a mean of 0.1 points (95% CI, −1.6 to 1.3 points) in the standard of care group, for a mean difference of 0.9 points (95% CI, −1.6 to 3.5 points; *P* = .46).

Thirteen participants (81.3%) in the VBAI group (95% CI, 53.7%-95.0%) achieved glycemic control and had a mean FBG level of less than 130 mg/dL (to convert glucose to millimoles per liter, multiply by 0.0555) at 8 weeks, reflecting maintenance of glycemic control; these 13 participants received 8.2 automated insulin titrations over 8 weeks. In the standard of care group, 4 participants (25.0%; 95% CI, 8.3%-52.6%) achieved glycemic control (difference, 56.3% [95% CI, 21.4%-91.1%]; *P* = .005). Of the 32 total participants, 30 (16 in the VBAI group and 14 in the standard of care group) had enough FBG data logged to be included in the analysis of mean change in FBG level. In the VBAI group, the mean (SD) FBG level decreased by 45.9 (45.9) mg/dL at 8 weeks (95% CI, −70.4 to −21.5 mg/dL) compared with a mean (SD) increase of 23.0 (54.7) mg/dL (95% CI, −8.6 to 54.6 mg/dL) in the standard of care group (difference, −68.9 mg/dL [95% CI, −107.1 to −30.7 mg/dL]; *P* = .001). On average, participants in the VBAI group logged data on 50 of the 56 days they were followed up (89.3%). The 13 participants (81.3%) who achieved glycemic control logged data on 54 of 56 days (96.4%).

During the 8-week trial period, the VBAI group had a mean (SD) of 1.3 (1.2) visits per participant with a diabetes-related health care professional compared with 1.3 (1.3) visits per participant in the standard of care group. In addition, 4 of 16 participants in the VBAI group had their protocol adjusted once by their clinician using our custom web portal; 3 instances were to change protocol parameters, including the target FBG range and the maximum allowed dose, and 1 instance was a dose increase. This dose change was not counted in the calculation of the mean number of autonomous insulin dose adjustments reported for the VBAI group. In the VBAI group, 11 of 16 participants had no contact with the research team after enrollment. The remaining 5 participants had contact 1 to 5 times with the research team via text message or email to address technical questions (3 participants with 1 contact, 1 participant with 2 contacts, and 1 participant with 5 contacts).

There were no adverse events requiring clinician intervention or participant withdrawal in this study. In the VBAI group, there were 11 episodes of nonsevere hypoglycemia that were all autonomously handled by the VBAI with insulin dose reductions. There were 10 episodes of nonsevere hypoglycemia logged in the standard of care group. There were no episodes of severe hypoglycemia or hyperglycemia in either group.

## Discussion

In this randomized clinical trial, we demonstrated the effectiveness of a VBAI in managing basal insulin titration compared with standard of care. Participants in the VBAI group had significantly faster insulin dose optimization, improved insulin adherence and glycemic control, and decreased diabetes-related emotional distress compared with those in the standard of care group. To our knowledge, this study marks the first time a VBAI has been used to autonomously adjust medication doses based on a protocol preapproved by a clinician. These findings suggest that digital health tools can be useful for medication titration and that voice user interfaces can be effective for patient-facing digital technologies.

Protocol adherence and frequent insulin titration were the primary drivers of rapid glycemic control in the VBAI group. To optimize adherence, the voice interface was designed to prioritize usability. Instructions were clear and simple. The daily check-in process was a multiturn conversation that took about 2 minutes to complete without requiring a smartphone or computer. On average, participants in the VBAI group logged data on 50 of the 56 days they were followed up (89.3%). The 13 participants (81.3%) who achieved glycemic control logged data on 54 of 56 days (96.4%). As a result, despite having strict blood glucose and insulin adherence criteria for titration, our VBAI was able to provide frequent titration recommendations. On average, participants in the VBAI group received 7.3 automated insulin titrations over 8 weeks, with the 13 who achieved glycemic control receiving 8.2 titrations. This finding further supports the benefit of frequent insulin titration to effectively achieve glycemic control. The willingness of participants to follow the VBAI’s instructions and the positive survey results regarding attitudes toward health technology more broadly suggest that patients might be accepting of this model of care delivery. In contrast, participants in the standard of care group received a mean of 1.6 titrations over 8 weeks and had a mean of only 1.3 clinic visits, which represented their only opportunity to receive diabetes care during the trial. They also had significantly lower insulin adherence. These factors possibly explain the increase in the mean FBG level seen in this group, as their insulin regimen was still inadequately optimized.

Medication compliance is a complex behavioral process, requiring daily engagement from patients. Many approaches have been developed to support patients, from nurse-based remote patient management interventions to digital tools such as smartphone applications.^36^ Voice-based conversational artificial intelligence has the potential to improve access to technology-enabled care for patients with low digital literacy, while simultaneously enhancing engagement for all patients. Voice-based conversational artificial intelligence applications that accelerate the time to control can also help counteract the negative effects of clinical engagement attrition that can occur among patients with chronic diseases.^37^ Although voice user interfaces are now almost ubiquitous in consumer applications, in health care, the use of voice interfaces is limited mostly to clinician-facing applications for clinical note transcription.^38^ This study highlights the potential of patient-facing VBAI platforms to augment care delivery. Although this study shows excellent patient engagement in the VBAI group over the trial period, diabetes requires lifelong patient involvement, and several studies have shown that sustained patient engagement with digital health devices is challenging.^39,40^ Future work should include longer follow-up to assess the association of VBAI applications with prolonged adherence, engagement, and glycemic control.

The technical application studied here was the use of AI to provide a conversational interface with patients. The actual clinical protocols implemented using this technology were guideline-based approaches to insulin titration with approval or modification by the patient’s physician. The technology was not designed to let the AI independently decide the dose titration. Our effort was to create a digital health tool that allows for AI-assisted medication management without increasing clinician workloads. In contrast to remote patient monitoring, we consider this model of care delivery as remote patient intervention (RPI). Remote patient intervention solutions close the loop on remote patient monitoring data to provide real-time disease and medication management for patients based on physician-approved protocols. Autonomous insulin dosing guidance, as shown in this study, is an example of RPI. There are many potential applications of RPI, including titration of other diabetes medications and management of chronic conditions (such as hypertension and heart failure). With this approach, we envision a future state of precision medicine in which clinicians approve medication protocols and AI assistants help patients execute these protocols at home. We believe that RPI solutions that incorporate voice-based AI applications can be particularly engaging, especially with the recent advancements in large language models and generative AI.^41^ As the field of digital health evolves to address the burden of diabetes and other chronic diseases, solutions that support innovative care models may be uniquely poised to improve clinical outcomes.

### Limitations

This study has many limitations. First, because participants were followed up for 8 weeks, glycemic control was measured by mean FBG level, rather than HbA_1c_ level. Second, it was not possible to determine whether incomplete insulin logs in the standard of care group reflected nonadherence to insulin or nonadherence to the log. However, this limitation was also present in the VBAI group, given that insulin adherence was similarly based on self-reported data. Third, incomplete FBG logs for some standard of care group participants necessitated review of medical records to determine whether glycemic control had been achieved at 8 weeks. Fourth, except for data collected during review of the medical record, all data collected in this study were self-reported. Fifth, this study does not compare VBAI with other apps using similar insulin titration software to specifically assess the attribution of a voice-based interface. Sixth, mean FBG levels worsened in the standard of care group, which could have led to an overestimate of the effect size of our intervention. Seventh, this study randomized only 39 English-speaking participants. Further work is needed to validate this technology in larger, more diverse populations.

## Conclusions

This randomized clinical trial found that a VBAI that provided autonomous basal insulin titration improved time to optimal insulin dosing, insulin adherence, and glycemic control among adults with type 2 diabetes compared with standard of care.

## Notes

Supplement 1.
Trial Protocol

Supplement 2. **eAppendix 1.** PAID-5 Survey **eAppendix 2.** Attitudes Toward Medication Adherence Survey **eAppendix 3.** Attitudes Toward Health Technology Survey **eMethods.** Voice-Based Conversational AI Titration Protocols **eFigure 1.** Physician Portal: Sending a Prescription to the Voice AI **eFigure 2.** Physician Portal: Viewing Titration History **eFigure 3.** Physician Portal: Viewing Blood Glucose Logs **eFigure 4.** Physician Portal: Viewing Insulin Logs **eAppendix 4.** Standard of Care Group Blood Glucose and Insulin Log

Supplement 3.
Data Sharing Statement
