# Supplementary material for: Use of Voice-Based Conversational Artificial Intelligence for Basal Insulin Prescription Management Among Patients With Type 2 Diabetes: A Randomized Clinical Trial
Source: JAMA Netw Open. 2023 Dec 1;6(12):e2340232. doi: 10.1001/jamanetworkopen.2023.40232 (PMC10692866; doi:10.1001/jamanetworkopen.2023.40232)
Supplement: Supplement 1. — Trial Protocol [file jamanetwopen-e2340232-s001.pdf]

SUPPLEMENT 1. Trial Protocol

Protocol Title: Using a voice assistant for insulin management in patients with diabetes

IRB Number: 58276

PI: Kevin Schulman

Approval date: 02-Feb-2021

Table of Contents

1. Purpose *2*

2. Background *2*

3. Overall Study Design *2*

3.1 Study design 2

3.2 Study population 2

3.3 Inclusion criteria 2

3.4 Exclusion criteria 3

4. Study Procedures *3*

4.1 Screening 3

4.2 Recruitment 3

4.3 Device setup 4

4.4 Intervention group 4

4.5 Control group 4

4.6 Data collection 4

4.7 Data analysis 5

5. Outcomes *5*

5.1 Primary outcomes 5

5.2 Secondary outcomes 6

6. Statistical Analysis Plan *6*

6.1 Power and sample size calculation 6

6.2 Analysis of results 7

7. Risks and Benefits *7*

7.1 Risk to participants 7

7.2 Risk of Amazon Echo Dot 7

7.3 Risk of insulin titration algorithm 7

7.4 Benefit to participants 8

8. Privacy and Confidentiality *8*

8.1 Privacy protections **8**
8.2 Audio data **8**
8.3 Confidentiality protections **8**
9. Summary of Protocol Revisions ***9***
9.1 Revision on 4/11/2021 .... **9**
9.2 Revision on 8/10/2021 .... **9**
9.3 Revision on 3/2/2022 .... **9**
9.4 Revision on 6/20/2022 .... **9**
10. References ***9***

1. Purpose

The purpose of this study is twofold: (1) To evaluate the feasibility of a digital voice assistant to help patients manage insulin-dependent diabetes between routine clinic visits. Specifically, the custom voice AI that we will develop will help patients log their blood sugars and medication use, as well as help them adjust their insulin dose based on a protocol that their physician prescribes. (2) To evaluate the effects of such an intervention on specific medical outcomes, such as medication adherence, blood sugar control and time to optimal insulin dose, as well as patient self-efficacy measures. The digital voice assistant used will be an Amazon Echo Dot and it will use custom voice AI software to help patients manage their diabetes at home.

We hope to learn whether a digital voice assistant can be reliably used by patients at home between clinic visits to improve management of insulin-dependent diabetes. We hope that this improved management will translate into improved medical outcomes. In the future, the findings of this study could serve as a first step towards developing an integrated outpatient solution that leverages voice assistant technology in the patient's home to improve management of multiple chronic medical conditions.

2. Background

There is a large amount of literature showing how digital health interventions can lead to improved clinical outcomes. In the area of voice assistant for use in diabetes care management, studies have shown that interactive voice responses through voice recognition software have the potential to improve medication and lifestyle modification adherence in diabetes and improve clinical outcomes. For example, in a randomized control trial of adults with diabetes, patients who received automated, interactive phone-delivered management interventions had greater decreases in hemoglobin A1c compared to standard of care.[1] Another randomized crossover study reported on a smartphone application that can synthesize verbal descriptions of meals and accurately return the corresponding insulin bolus dose.[2] A similar voice-activated device was identified in calculating insulin dosages in the visually impaired with diabetic retinopathy.[3] Ultimately, no study has yet embarked on what this study looks to investigate; however, a review of key leading opinions from clinical, engineering, and data security experts have identified smart devices, voice assistants and the Internet of medical things (IoMT) as a key untapped area of potential for optimizing the home health care ecosystem to reduce diabetic complications, including diabetic foot ulcers.[4]

3. Overall Study Design

3.1 Study design

This is a prospective, single-center, fully remote (decentralized), randomized, open-label, parallel-group trial.

3.2 Study population

We will include adults with a diagnosis of type 2 diabetes with need for initiation or adjustment of a long-acting insulin regimen.

3.3 Inclusion criteria

1) Age ≥ 18 years

2) Established diagnosis of type 2 diabetes
3) Requiring initiation or adjustment of once-daily basal insulin

3.4 Exclusion criteria
1) Patients on insulin pumps
2) Patients with impaired decision-making capacity
3) Patients who do not speak English
4) Patients who do not own a smartphone or have access to a WiFi network at home

4. Study Procedures

4.1 Screening

Our team will recruit patients with type 2 diabetes who have been recently started on insulin (or who are having active adjustments to their insulin regimen). To identify patients that may be eligible for recruitment, our team will use Stanford's STARR cohort discovery tool and an Epic workbench report overseen by the Stanford Population Health Medical Director. We will use these tools to create a report of patients at Stanford Internal Medicine and Endocrinology clinics with type 2 diabetes and a recent hemoglobin A1c greater than 8%. Specifically, this is done through a cohort discovery of patients who have seen providers who practice at the Stanford primary care and Endocrinology clinic sites. In addition, we will also be identifying patients through advertisements through the Stanford Diabetes Research Core. Specifically, the recruitment director with the core will be giving us access to a listserv of Stanford-affiliated patients who have requested to be contacted about diabetes-related clinical trial opportunities.

For potential participants identified through STARR or Epic workbench reports, our team will first contact the primary care providers and/or Endocrinologists of these patients (depending on which providers are managing or co-managing the patient's diabetes regimen). If the primary care providers and/or Endocrinologists agree that their patient might benefit from inclusion in our study, a member of the patient's care team will first contact the patient to notify them of the study and obtain approval for initiation of the research team's enrollment process. Upon approval, our research team will reach out to provide more information and conduct the informed consent process if the patient would like to participate.

For potential participants identified through the SDRC listserv, if these participants directly respond to our listserv advertisement, our research team will reply back to them directly. Our team will also contact their primary care providers and/or endocrinologists if the potential participant decides to enroll in the trial.

4.2 Recruitment

When an eligible candidate is identified per the screening process above, the research team will contact the patient's primary care physician and inform them of this study. If the patient's primary care physician agrees that the patient would benefit from inclusion in this study, they will reach out to the patient and describe the trial to them. If the patient agrees to be contacted by the research team, their care team member will share their information with a member of the research team via secure message and a member of the team will reach out via the participant's preferred method of communication (phone, email, etc). Due to the COVID-19 pandemic and the fact that diabetes is a risk factor for the virus, all participants will undergo the informed consent process remotely. The research team will make contact with the participant via the mechanism shared with them by the treating team, introduce the project and ask if the participant would like to undergo the informed consent process via an online REDCap survey or via a paper consent form sent in the mail. The research team member will then send the Informed Consent Form (ICF) to the participant via either a URL by e-mail or a paper ICF by mail. The research team member will set up a time to discuss the ICF with the participant either by phone or video chat and will describe the study to the participant, review the risks and benefits outlined in the study consent form and address any questions from the participant before asking them for their consent to participate in the study. After consent is obtained, participants will complete an intake survey and will be randomized to either the intervention or control group. All participant information obtained during enrollment will be stored securely in a HIPAA-compliant and PHI-safe Stanford Medicine Box folder.

4.3 Device setup

After consent is obtained, the participant's treating physician will communicate their desired insulin protocol with the research team. The information communicated will include the following:

- Starting insulin prescription
- Goal fasting blood sugar
- Titration parameters (e.g. Increase starting dose by 2 units every 3 days if not at goal fasting blood sugar)
- Situations in which they would want their patient to contact their provider or seek immediate medical attention and call 911/report to an ER

This information will be communicated with the research team via either a custom Epic order set currently in development or a secure online portal developed by the research team. The provider will only have to provide the patient's unique study ID in order to enter this information in the portal. This ID will be securely emailed to the provider upon enrollment of the patient. The provider will be asked to confirm their prescription inputs via the secure online portal to reduce the probability of erroneous data insertion. The provider will also have to confirm the ID of the patient to prevent the provider from erroneously placing a prescription for the wrong patient. No identifying information will be collected by the portal. For participants in the intervention group, this information will be programmed into the digital voice assistant application. For participants in the control group, this information will be recorded for future data analysis.

4.4 Intervention group

Due to the COVID-19 pandemic and the fact that diabetes is a risk factor for the virus, we will mail devices to participants in the intervention group. We will also provide them with the option to set up a phone call or video chat with a member of the research team to help them with device set up.

Device setup will include the following steps:

- Open Alexa Echo Dot package and plug in to power
- Downloading the "Alexa" smartphone application
- Through the Alexa smartphone application, enabling our custom application (called an "Alexa Skill").
- Logging in to our custom application with a unique study ID that is linked to the participant's insulin protocol.

A detailed document called "Alexa Setup Instructions" will be provided to subjects that goes into granular detail on how to set up the device as well as its functionalities. The entire set up process should take about 5-10 minutes.

Participants in the intervention group might also be given a blood sugar and medication administration log and be asked to use both the voice assistant and the blood sugar and medication administration log for 2 of the 8 weeks they are participating in the trial.

4.5 Control group

For participants in the standard of care control group, we will email them a copy of a blood sugar and medication administration log for them to fill out electronically through REDCap or print and fill out. If a participant doesn't have access to a printer, we will mail them a copy of the log. All participants in the control group will also receive an Amazon Echo Dot, and the research team will help participants set a daily alarm on the device to remind participants to log their blood sugar and insulin administration data on their REDCap log. Participants in the control group will NOT have access to the custom Alexa skill and will not be using the device to log data or receive dose adjustment instructions.

4.6 Data collection

Data will be collected daily from participants in the intervention and control groups of the study. Participants in the intervention group will be able to interact with our custom voice AI by speaking with their Echo Dot. Participants will be instructed to "check in" with the AI once daily at which point the AI will collect information about recently checked blood sugars and recent administration of insulin. Data collected by the device will be read back to the patient for confirmation. At the end of the check in, the voice AI will remind patients about the details of their insulin regimen. Based on the patient's self-reported data, this reminder might include a prompt to change the dose of their insulin. All reminders and prompts will be strictly based on the physician-prescribed insulin protocol at the

beginning of the study, and prompts will include a summarization of the rationale behind the recommendation (e.g. "Your fasting blood sugars are still above goal. Based on your prescribed treatment plan, you should increase your dose of Lantus to 17 units."). No automated decisions will be made by the voice assistant. The voice assistant will only be assisting the patient with instructions that patients are normally given in writing by their provider. An instruction manual will be provided to the patient with details on how to interact with the device. The only data collected from the patient will be patient-reported blood sugar data and medication administration occurrences. The audio data collected by the device is encrypted and sent to the Amazon cloud for processing into relevant text. The text data is supplied to our application for analysis and storage. Our application will not receive or store any audio data. No protected health information will be collected by our software. All data will be de-identified and stored in the Amazon Web Services cloud. All data will be linked to a participant's unique study ID. A document linking a participant's unique ID to their name and medical record number will be stored on the secure PHI-safe Stanford Medicine Box server. Patients in the control group will use their provided log, labeled with their unique study ID, to record their daily blood glucose checks and insulin adherence. This is part of the standard of care in patients with insulin-dependent diabetes. At the end of the trial period, we will collect this log from patients for data analysis. Participants can choose to securely email or upload this log to the research team through REDCap, or if they prefer, they will be provided with a pre-paid envelope to mail the log free of cost. Finally, user satisfaction survey data will be collected at the beginning and end of the study from patients in the intervention and control groups. The first survey will include "Problem Areas in Diabetes" ("PAID") questions validated in the literature as a rapid screen for diabetes-related emotional distress. The second survey will include questions specifically tailored towards interrogating the patient's attitude toward and comfort with insulin dose adjustments ("Medication Adherence Survey"). The third and final survey will include questions on attitudes towards healthcare technology ("Health Technology Survey"). All surveys will be collected online via REDCap.

4.7 Data analysis

Members of the research team will review the collected blood sugar and insulin administration data for analysis. Specifically, we will analyze the collected blood sugar data to determine how often blood sugars were checked and how well-controlled they were. We will also analyze insulin administration data to determine how adherent participants were to their prescribed regimen and how effectively participants were able to titrate their insulin to an effective dose. We will also analyze study survey data to assess participant's diabetes self-efficacy and attitudes towards healthcare technology. In addition to reviewing data collected by our app and REDCap, the research team will also access the medical record of participants who have completed the trial to obtain certain lab results and medication data to help supplement any missing data from the trial. Specifically, the research team will obtain recent blood sugar and Hemoglobin A1c values, as well as the most recent insulin prescription. This will help the research team perform data analysis in cases where trial data are incomplete. Please note that this is only for participants who have been consented, enrolled and have completed the full 8 weeks of the trial. If participants decide to withdraw from the study, they can do so at any time by contacting Ashwin Nayak at 650-308-8062 or aknayak@stanford.edu. After study completion, participants will be able to keep their Amazon Echo device for personal use. Their specific login ID will be disabled which will preclude them from using our custom application after study completion. If participants decide to withdraw from the study before completion, they will be asked to return their Amazon Echo device. They will be provided with pre-paid packaging to do so.

5. Outcomes

5.1 Primary outcomes

1) Time to Optimal Insulin Dose
    a. Measured as the number of days between the study start date and the date that goal 3-day mean fasting blood glucose is achieved
2) Mean Insulin Medication Adherence
    a. Measured as the percentage of days during the study period with a logged insulin dose
3) Change in Composite Score of Attitudes Toward Diabetes (PAID-5)
    a. This survey contains 5 items. Each item contains 5 responses, scored 0-4.
    b. For this survey, higher scores indicate more diabetes-related emotional distress.
    c. The composite score is calculated as the sum across all 5 items (the minimum score is 0, the maximum score is 20).
    d. The change in composite score is calculated as:
        i. Δ = (Composite score at 8 weeks) – (Composite score at baseline)
        ii. A negative value for Δ reflects an **improvement** in diabetes-related emotional distress.

   e. The full survey is available in supplement 2.

4) Change in Composite Score of Attitudes Toward Health Technology
   a. This survey contains 2 items. Each item contains 5 responses, scored 0-4.
   b. For this survey, higher scores indicate more favorable attitudes toward voice assistant technology.
   c. The composite score is calculated as the sum across all 2 items (the minimum score is 0, the maximum score is 8).
   d. The change in composite score is calculated as:
      i. Δ = (Composite score at 8 weeks) – (Composite score at baseline)
      ii. A positive value for Δ reflects an **improvement** in attitudes toward voice assistant technology.
   e. The full survey is available in supplement 2.
5) Change in Composite Score of Attitudes Toward Medication Adherence
   a. This survey contains 5 items. Each item contains 5 responses, scored 0-4.
      i. Note that for Q2 and Q5, the scale is reversed, meaning that the response “Strongly Agree” is scored as 0 and the response “Strongly Disagree” is scored as 4.
   b. For this survey, higher scores indicate more favorable attitudes toward medication adherence.
   c. The composite score is calculated as the sum across all 5 items (the minimum score is 0, the maximum score is 20).
   d. The change in composite score is calculated as:
      i. Δ = (Composite score at 8 weeks) – (Composite score at baseline)
         1. Please note that Q4 and Q5 are not always answered at baseline, depending on if a patient is on an active insulin prescription. If Q4 and Q5 are not answered at baseline, the maximum survey score is 12 and Q4 and Q5 are ignored when calculating the composite score at 8 weeks.
      ii. A positive value for Δ reflects an **improvement** in attitudes toward medication adherence.
   e. The full survey is available in supplement 2.

5.2 Secondary outcomes

1) Glycemic Control
   a. Measured as the proportion of participants who achieve goal 3-day mean fasting blood glucose by 8 weeks.
   b. In the control arm, if blood glucose data logs are incomplete, the participant’s medical record will be reviewed for evidence of achieving glycemic control.
2) Glycemic Improvement
   a. Measured as the change in 3-day mean fasting blood glucose values from baseline to 8 weeks.
   b. Calculated as:
      i. Δ = (3-day mean fasting blood glucose at 8 weeks) – (3-day mean fasting blood glucose at baseline)
   c. If fasting blood glucose logs are incomplete in the final 3 days of the 8-week period, the last 3 fasting blood glucose values will be used provided they are from the last 2 weeks of the 8-week period.
   d. If fasting blood glucose logs are fully absent in the final 2 weeks of the 8-week period, the participant’s medical record will be reviewed for evidence of fasting blood sugar values.
      i. To qualify as a fasting blood sugar value, there must be clinician documentation that a value was fasting. Lab values without interpretation cannot be assumed to be fasting.

6. Statistical Analysis Plan

6.1 Power and sample size calculation

Sample size was determined to be 32 participants for 80% power and a 2-sided alpha of 0.05 to detect a treatment difference of 84% for the voice AI group vs 40% for the standard of care group for the proportion that achieve glycemic control. We decided to use our secondary outcome of glycemic control as the basis of our power calculation because it has been used in prior studies.

The treatment difference of 84% vs 40% was based on a similar study which found a 2:1 (60% vs 30%) treatment difference for their digital intervention.[5] Given that this similar study supported titration of more complicated insulin regimens and did not utilize voice, we felt that an increased projected treatment effect of 84% for our intervention was reasonable. We also requested and received approval to recruit up to 50 participants to account for drop out.

6.2 Analysis of results

The primary outcome, time to optimal insulin dose, will be assessed using time to event analyses: Kaplan Meier curves and the log rank test to compare the voice AI intervention and standard of care control. Insulin adherence, change in attitudes (toward diabetes, technology, and medical adherence), and glycemic improvement (change in 3-day mean fasting blood glucose) will be compared using the Student's two sample t-test of means. The two-sample test of proportions will be used to compare glycemic control for the voice AI intervention and standard of care control. The Holm-Bonferroni correction will be applied to the standard $\alpha = 0.05$ Type I error rate to adjust for multiple comparisons.[6]

7. Risks and Benefits

7.1 Risk to participants

This study poses minimal risk to its participants. Patients in the control group will receive standard of care management of insulin-dependent diabetes. Patients in the intervention arm will not be subject to any risky treatments. They will be interacting with a digital tool that will collect information and remind them of their physician-prescribed treatment plan. The digital tool will not make any autonomous medical decisions or alter the physician's plan in any way. All data collected by the digital tool is anonymized. The voice assistant device will confirm the patient's self-reported data with readback to the subject and verbal confirmation by the subject, prior to storing the data. If a blood sugar reading is outside a standard safe range as determined by their physician, the voice assistant device will provide them the same instructions that would be provided on written instructions by their provider. For example, if a patient's reported blood sugar is less than 70, the voice assistant may notify the subject that their doctor has instructed at such blood sugars to consume something sweet and recheck their sugar. If a patient's reported blood sugar is greater than 350, the voice assistant may notify the patient that their doctor has instructed at such blood sugars to notify a medical professional immediately. If a patient's sugars are dangerously hypoglycemic or dangerously hyperglycemic as determined by their provider, the device will inform the patient to seek medical attention immediately and report to an ER/call 911 per their provider's instructions.

The risks of participating in this study include the possibility of inaccurate self-reporting by participants leading to improper insulin dose adjustments. As stated above, our application will confirm all self-reported data prior to logging it by reading back reported values to patients and requiring verbal confirmation from the patient. Furthermore, all dose adjustments will be small (ranging from 2-4 units based on the participant's prescription). Such small dose adjustments to long-acting insulin are very unlikely to cause major adverse events such as hypoglycemia.

7.2 Risk of Amazon Echo Dot

This is a non-significant risk device because it doesn't pose a serious risk to health, safety or welfare of the subject. Its use is not of substantial importance in diagnosing, curing or treating disease.

7.3 Risk of insulin titration algorithm

PCPs routinely provide patients with instructions on how to titrate their insulin at home. This algorithm helps patients implement these instructions (which is something they would otherwise be doing with no assistance). We believe that the software poses non-significant risk for the following reasons:

1) The provider will be able to set a limit to how much and how often the algorithm will increase the insulin dose.
2) The provider will be able to set a maximum insulin dose.
3) The default insulin titration protocol that we provide mirrors the 2020 consensus statement from AACE (American Association of Clinical Endocrinologists) and ACE (American College of Endocrinology) guidelines, but is more conservative to have less tolerance for hypoglycemic events

4) In partnership with Endocrinologists and as Internists ourselves, we have a well thought out, professional guideline-based, conservative hypoglycemia protocol that reduces insulin dose and automatically turns off the titration algorithm if needed to account for various clinical situations where hypoglycemia may be present. The protocol is also designed to account for when providers may have erroneously started too high an insulin dose.
5.) There are multiple steps for confirmation when reporting data to ensure correct data acquisition, including reminders with every single check in that a "fasting blood sugar" is one where one has not had anything to eat in the 8 hours prior.

7.4 Benefit to participants

There are no guaranteed benefits to be gained by the participants. However, ideally, patients in the intervention arm will benefit from increased engagement with their care, improved medication adherence and improved perceptions of medical technology and home-based medical care. We also believe that in the future, this technology may lead to reduced ED visits, hospital admissions and diabetes-related complications include diabetic ulcers, diabetic retinopathy, diabetic nephropathy, and diabetic neuropathy.

8. Privacy and Confidentiality

8.1 Privacy protections

PHI will only be collected once during enrollment, which includes an entry survey on REDCap. The least amount of information will be obtained to accomplish the purpose of the research. This data will be stored securely in a folder on the HIPAA-compliant Stanford Medicine Box. A unique study ID will be randomly generated for each patient (not derived from any identifiable information). This ID will be used with our application and will be linked with the self-reported patient data that we collect using the Amazon Echo Dot. No PHI will be collected or stored by our application on the Amazon Echo Dot.

8.2 Audio data

Amazon Echo devices (and all similar voice assistant technologies) record audio during user interactions. This audio data is encrypted and sent to the Amazon cloud for processing into relevant text. The text data is supplied to our application for analysis and storage. Our application will not receive or store any audio data. All audio data collected by Amazon Echo devices are visible in the Alexa app for users to see and delete. Furthermore, Amazon explicitly states that its devices do not record audio data when users are not interacting with its devices. Whenever an Amazon Echo device is recording audio data, a special indicator light will turn on to alert the user. These details will be included in the informed consent process and patients will be instructed on how to delete audio recordings in their Amazon privacy settings if desired.

8.3 Confidentiality protections

Identifiers will be used to screen for and recruit patients and will be used throughout the consenting process. Identifiers will also be used when coordinating the mailing of a device and/or patient logs. Health information will be used to identify potential patients who meet our inclusion criteria of having a diagnosis of Type 2 Diabetes and have a requirement for starting or increasing long-acting insulin. We will be working with the following types of data in support of screening and recruitment activities:

- Full Name
- Addresses
- Medical record numbers
- Phone numbers
- Email addresses
- Demographics (age, sex, etc)
- Lab or test results
- Diagnosis or procedure codes
- Prescriptions or medications

All collected PHI and health identifiers will be collected during the screening or recruitment process and stored securely on Stanford Medicine Box in a PHI-safe folder. The data collected during the study by our custom application on the Amazon Echo Dot will be de-identified self-reported blood glucose and insulin administration data. This data will be encrypted and securely stored in the Amazon Web Services (AWS) cloud. Access to the data will be password protected and as stated earlier, no identifiable data will be stored on the AWS server. The AWS server will be protected by Secure Socket Layer (SSL) encryption. Informed consent and all study survey data will

be collected and managed using REDCap (Research Electronic Data Capture). REDCap is a secure, web application designed to support data capture for research studies. It provides user-friendly, web-based case report forms, real-time data entry validation, audit trails and de-identified data export mechanism. The system was developed by a multi-institutional consortium which includes Stanford University and was initiated at Vanderbilt University. The database is hosted at the Stanford University School of Medicine secure data center. The system is protected by Secure Socket Layer (SSL) encryption and a strong web-based authentication system.

9. Summary of Protocol Revisions

9.1 Revision on 4/11/2021

In the original protocol, we stated that we would be enrolling patients at Stanford Internal Medicine clinic, but we didn't specify which clinic or if we would use multiple sites. In this revision, we clarified that we would be enrolling patients at 4 different Stanford Internal Medicine clinic sites (Stanford Internal Medicine East, Stanford Internal Medicine Los Altos, Stanford Internal Medicine Santa Clara and Stanford Internal Medicine Portola Valley). We also added 5 Stanford Internal Medicine physicians to the IRB who work at these various sites to aid with participant enrollment, as well as two additional research administrators. We also made a small modification to the consent form regarding compensation for the study. To ensure equitable compensation for the control arm, we would be giving control participants an echo dot smart speaker as well.

9.2 Revision on 8/10/2021

Added an additional physician and Stanford clinic site (Stanford Endocrinology) to the IRB. Also added two additional recruitment channels at Stanford: 1.) A trial advertisement through a listserv overseen by the Stanford Diabetes Research Core, and 2.) Utilization of Epic Population Health reporting tools to identify potential study candidates.

9.3 Revision on 3/2/2022

Modified 1 of the exclusion criteria to make it more specific. Previously, this criterion stated that we would exclude "patients who are on complicated insulin regimens (multiple insulins, insulin pump, etc)". This was updated to exclude "patients who are on insulin pumps". We had noticed that there were many candidates who might benefit from this trial but were excluded due to being on more than 1 type of insulin. With this change, a patient who is on both long-acting and short-acting insulin would now be able to participate in the trial if they needed adjustment in long-acting insulin.

9.4 Revision on 6/20/2022

We requested access to the medical records of enrolled participants who had completed the trial in order to obtain certain lab results and medication data to help supplement any missing data from the trial. Specifically, we requested access to recent blood sugar and Hemoglobin A1c values, as well as the most recent insulin prescription.

10. References

1. Williams ED, Bird D, Forbes AW, et al. Randomised controlled trial of an automated, interactive telephone intervention (TLC Diabetes) to improve type 2 diabetes management: baseline findings and six-month outcomes. *BMC Public Health*. 2012;12:602. Published 2012 Aug 3. doi:10.1186/1471-2458-12-602
2. Foltynski P, Ladyzynski P, Pankowska E, Mazurczak K. Efficacy of automatic bolus calculator with automatic speech recognition in patients with type 1 diabetes: A randomized cross-over trial. *J Diabetes*. 2018;10(7):600-608. doi:10.1111/1753-0407.12641
3. Radfar M, Hamilton M, Ming A. A voice activated device for insulin dosage calculations for visually impaired diabetes. *2011 24th Canadian Conference on Electrical and Computer Engineering(CCECE).* 2011; 000904-000907
4. Basatneh R, Najafi B, Armstrong DG. Health Sensors, Smart Home Devices, and the Internet of Medical Things: An Opportunity for Dramatic Improvement in Care for the Lower Extremity Complications of Diabetes. J Diabetes Sci Technol. 2018;12(3):577-586. doi:10.1177/1932296818768618
5. Bergenstal RM, Johnson M, Passi R, et al. Automated Insulin Dosing Guidance to Optimize Insulin Management in Patients with Type 2 Diabetes; A Multi-Center Randomized-Controlled Trial. Lancet. 2019;393(10176):1138-1148. doi:10.1016/S0140-6736(19)30368-X
6. Holm S. A Simple Sequentially Rejective Multiple Test Procedure. *Scandinavian Journal of Statistics*. 1979;6(2):65-70.
