# Supplementary material for: Use of Voice-Based Conversational Artificial Intelligence for Basal Insulin Prescription Management Among Patients With Type 2 Diabetes: A Randomized Clinical Trial
Source: JAMA Netw Open. 2023 Dec 1;6(12):e2340232. doi: 10.1001/jamanetworkopen.2023.40232 (PMC10692866; doi:10.1001/jamanetworkopen.2023.40232)
Supplement: Supplement 2. — eAppendix 1. PAID-5 Survey eAppendix 2. Attitudes Toward Medication Adherence Survey eAppendix 3. Attitudes Toward Health Technology Survey eMethods. Voice-Based Conversational AI Titration Protocols eFigure 1. Physician Portal: Sending a Prescription to the Voice AI eFigure 2. Physician Portal: Viewing Titration History eFigure 3. Physician Portal: Viewing Blood Glucose Logs eFigure 4. Physician Portal: Viewing Insulin Logs eAppendix 4. Standard of Care Group Blood Glucose and Insulin Log [file jamanetwopen-e2340232-s002.pdf]

Supplementary Online Content

Nayak A, Vakili S, Nayak K, et al. Use of voice-based conversational artificial intelligence for basal insulin prescription management among patients with type 2 diabetes: a randomized clinical trial. *JAMA Netw Open.* 2023;6(12):e2340232. doi:10.1001/jamanetworkopen.2023.40232

eAppendix 1. PAID-5 Survey
eAppendix 2. Attitudes Toward Medication Adherence Survey
eAppendix 3. Attitudes Toward Health Technology Survey
eMethods. Voice-Based Conversational AI Titration Protocols
eFigure 1. Physician Portal: Sending a Prescription to the Voice AI
eFigure 2. Physician Portal: Viewing Titration History
eFigure 3. Physician Portal: Viewing Blood Glucose Logs
eFigure 4. Physician Portal: Viewing Insulin Logs
eAppendix 4. Standard of Care Group Blood Glucose and Insulin Log

This supplementary material has been provided by the authors to give readers additional information about their work.

© 2023 Nayak A et al. *JAMA Network Open.*

eAppendix 1. PAID-5 Survey

Start of Block: PAID-5

Instructions: Which of the following diabetes issues are currently a problem for you? Select the response that gives the best response for you.

Q1. Feeling scared when you think about living with diabetes.

- ○ Not a Problem
- ○ Minor Problem
- ○ Moderate Problem
- ○ Somewhat Serious Problem
- ○ Serious Problem

Q2. Feeling depressed when you think about living with diabetes.

- ○ Not a Problem
- ○ Minor Problem
- ○ Moderate Problem
- ○ Somewhat Serious Problem
- ○ Serious Problem

Q3. Worrying about the future and the possibility of serious complications.

- ○ Not a Problem
- ○ Minor Problem
- ○ Moderate Problem
- ○ Somewhat Serious Problem
- ○ Serious Problem

Q4. Feeling that diabetes is taking up too much of your mental and physical energy every day.

- ○ Not a Problem
- ○ Minor Problem
- ○ Moderate Problem
- ○ Somewhat Serious Problem
- ○ Serious Problem

Q5. Coping with complications of diabetes.

- ○ Not a Problem
- ○ Minor Problem
- ○ Moderate Problem
- ○ Somewhat Serious Problem
- ○ Serious Problem

End of Block: PAID-5

© 2023 Nayak A et al. *JAMA Network Open.*

eAppendix 2. Attitudes Toward Medication Adherence Survey

Start of Block: Attitudes Toward Medication Adherence

The next 5 questions ask about diabetes medication adherence. Please select your level of agreement with the statements below.

Q1. I have confidence in my ability to increase my insulin dose as instructed by my doctor.

- Strongly Agree
- Somewhat Agree
- Neither Agree nor Disagree
- Somewhat Disagree
- Strongly Disagree

Q2. Figuring out what dose of insulin to take is confusing.

- Strongly Agree
- Somewhat Agree
- Neither Agree nor Disagree
- Somewhat Disagree
- Strongly Disagree

Q3. It is easy to remember to take my insulin on time.

- Strongly Agree
- Somewhat Agree
- Neither Agree nor Disagree
- Somewhat Disagree
- Strongly Disagree

Q4. Please answer only if on an active insulin prescription: I check my blood sugars and take my insulin as prescribed every day.

- Strongly Agree
- Somewhat Agree
- Neither Agree nor Disagree
- Somewhat Disagree
- Strongly Disagree

Q5. Please answer only if on an active insulin prescription: I may have accidentally taken the wrong dose of insulin by mistake.

- Strongly Agree
- Somewhat Agree
- Neither Agree nor Disagree
- Somewhat Disagree
- Strongly Disagree

End of Block: Attitudes Toward Medication Adherence

© 2023 Nayak A et al. *JAMA Network Open.*

eAppendix 3. Attitudes Toward Health Technology Survey

Start of Block: Attitudes Toward Health Technology

The last questions ask about your attitudes toward health technology. Please select your level of agreement with the statements below.

Q1. I feel comfortable using a virtual voice assistant (e.g. Amazon Alexa, Apple Siri, Google Assistant) to assist me with my diabetes care.

- ○ Strongly Agree
- ○ Somewhat Agree
- ○ Neither Agree nor Disagree
- ○ Somewhat Disagree
- ○ Strongly Disagree

Q2. I feel comfortable using a virtual voice assistant (e.g. Amazon Alexa, Apple Siri, Google Assistant) to assist me with my insulin dose adjustments.

- ○ Strongly Agree
- ○ Somewhat Agree
- ○ Neither Agree nor Disagree
- ○ Somewhat Disagree
- ○ Strongly Disagree

End of Block: Attitudes Toward Health Technology

© 2023 Nayak A et al. *JAMA Network Open.*

eMethods. Voice-Based Conversational AI Titration Protocols

Insulin Titration Protocols

Default prescription parameters (all customizable unless specifically noted):

- Goal Fasting Blood Glucose Range: 90 – 130
- Starting insulin dose: 0.2 U/kg (e.g. 15 units) (conservative protocol: 0.1 U/kg)
- Maximum permitted dose: 0.6 U/kg (e.g. 45 units) (conservative protocol: 0.5 U/kg)
- Insulin dose frequency: QAM
- Titration Schedule:
    - FBG 130-139, add 1U, FBG 140-180, add 10%, FBG >180, add 20% (conservative protocol: FBG greater than goal, add 2U)
    - FBG below goal but not considered "hypoglycemic event", decrease by 2U
    - Adjustment period (how often dose is adjusted): Glargine→ q3 days, Toujeo/Tresiba→ q5 days
- Minimum titration requirements (these parameters must be met for the dose to be adjusted. These are NOT customizable):
    - Patient must check and report their fasting blood glucose: Every day for the adjustment period
    - Patient must report their insulin dose: Every day for the adjustment period

An example using the default parameters:
If your patient weighs 100kg, the patient starts at 20 units of Glargine QAM (0.2U/kg). They must report their fasting blood glucose and the insulin dose they took each day for 3 days in a row for the voice app to adjust their dose. If their average fasting blood sugar over those 3 days is above 180, they will be instructed to take 24 units QAM (+20%). If their average fasting blood sugar is below 90, they will be instructed to take 18 units QAM (-2U). If they did not log the required data for 3 days in a row, they will be reminded to log their data and no dose adjustments will be made until they meet 3 days in a row of logged data. If they have a "hypoglycemic event", their insulin will be adjusted per the hypoglycemia protocols noted in the next section.

If a patient records taking a dose of insulin that is not their prescribed dose, at the next adjustment period the protocol will take this into account to safely adjust their dose based on what they have reported taking instead of their prescribed dose. For example, your patient is prescribed 10 units of Glargine QAM. For the 3 days in the adjustment period, their average fasting blood sugar is within goal but they report taking 8 units one night, then 9 units, then 8 units. The protocol will instruct them to take 9 units, the maximum dose they actually took that period. However, if patients report taking some insulin doses that are both above their prescription and below their prescription within the same time period, then the protocol will recommend no dose change.

Hypoglycemia Protocols
By default, patients are told to inform their provider any time a hypoglycemic episode takes place.

When the insulin titration protocol is turned off, the device will not make any dose adjustments until the patient's PCP chooses to turn the protocol back on.

Severe hypoglycemia is defined by default as BG <40. If a severe hypoglycemia event occurs at any time, the patient is told to eat something sweet and seek immediate medical attention. The insulin titration protocol turns off.

Non-severe hypoglycemia is split into two groups as follows:

- Moderate non-severe hypoglycemia by default as BG 40-69
- Mild non-severe hypoglycemia by default as BG 70-85 with symptoms of hypoglycemia

If a patient experiences a non-severe hypoglycemia event, the patient is told to eat something sweet and recheck their blood sugar until their blood sugar is >100 or they no longer have symptoms. If on repeat check they still have non-severe hypoglycemia with a non-fasting BG, the patient is told to eat something sweet again and recheck their BG again. If on third recheck within that day their non-fasting BG is still non-severely hypoglycemic, the patient is told to eat something sweet, report to the ER and the insulin titration protocol turns off.

© 2023 Nayak A et al. *JAMA Network Open.*

If a patient reports moderate non-severe hypoglycemia, their next dose of insulin by default decreases by 25%. If they have already had a moderate non-severe hypoglycemia episode within the past 7 days, their next insulin dose by default decreases by 50%. If they have already had 2 moderate non-severe hypoglycemia episodes within the past 7 days, the insulin titration protocol turns off and the patient is told to report to their doctor for reassessment of their insulin protocol.

When a patient reports mild non-severe hypoglycemia, their next dose of insulin by default decreases by 10%. If they have already had 1 mild non-severe hypoglycemia episode within the past 7 days, their next insulin dose by default decreases by 25%. If they have already had 2 mild non-severe hypoglycemia episodes within the past 7 days, their next insulin dose by default decreases by 50%. If a fourth non-severe hypoglycemic episode happens within the past 7 days, the insulin titration protocol turns off and the patient is told to report to their provider for reassessment of their insulin protocol.

In situations where patients have both mild and moderate non-severe hypoglycemic episodes within the past 7 days, the protocol will look at the last dose reduction to inform the next dose reduction (-10%--> -25%--> -50%--> turn insulin protocol off).

Hyperglycemia Protocols
By default, if a reported BG is >350, the patient will be informed to contact their doctor immediately,

By default, if a reported BG >500, the patient will be informed to contact their doctor and seek immediate medical attention.

© 2023 Nayak A et al. *JAMA Network Open.*

eFigure 1. Physician Portal: Sending a Prescription to the Voice AI

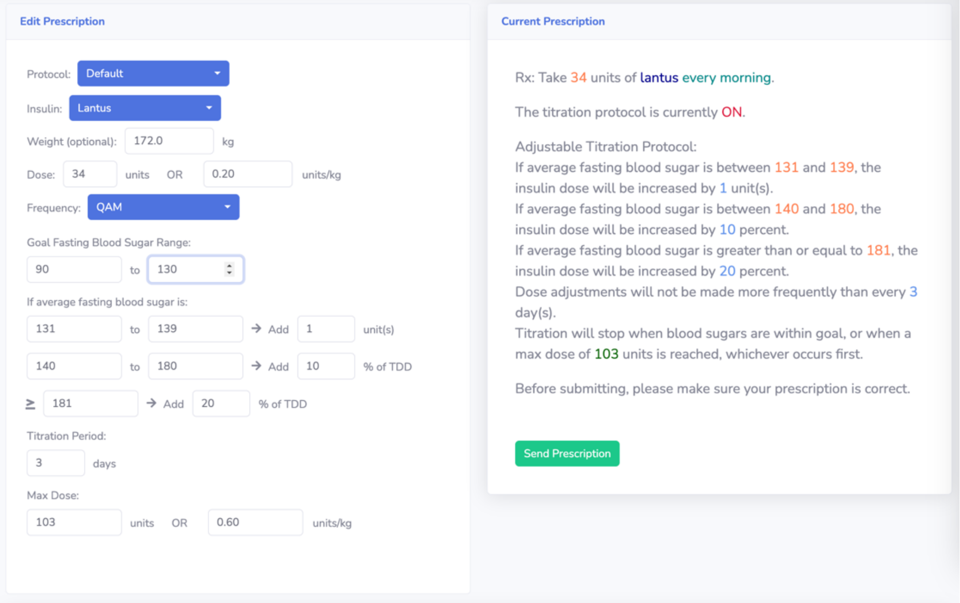

Providers use our custom web portal to send insulin titration protocols to our voice AI. Default parameters were pre-populated and customizable. The insulin protocol included the following parameters: insulin type, starting dose and frequency, goal fasting blood sugar range, titration ranges and amounts, titration period, and max dose.

Abbreviations: kg, kilograms; QAM, every day in the morning; TDD, total daily dose

© 2023 Nayak A et al. *JAMA Network Open.*

eFigure 2. Physician Portal: Viewing Titration History

Prescription History

Show 10 entries

Search:

Date	Time	Provider	Prescription	Titration Protocol	Status
2021-05-27	15:14:11	Alexa	Take 45 units of Basaglar every morning.	If average fasting blood sugar is between 131 and 139, the insulin dose will be increased by 1 unit(s) every 3 day(s). If average fasting blood sugar is between 140 and 180, the insulin dose will be increased by 10 percent every 3 day(s). If average fasting blood sugar is greater than or equal to 181, the insulin dose will be increased by 20 percent every 3 day(s). Titration will stop when blood sugars are within goal, or when a max dose of 88 units is reached, whichever occurs first.	Active
2021-05-23	09:38:45	Alexa	Take 44 units of Basaglar every morning.	If average fasting blood sugar is between 131 and 139, the insulin dose will be increased by 1 unit(s) every 3 day(s). If average fasting blood sugar is between 140 and 180, the insulin dose will be increased by 10 percent every 3 day(s). If average fasting blood sugar is greater than or equal to 181, the insulin dose will be increased by 20 percent every 3 day(s). Titration will stop when blood sugars are within goal, or when a max dose of 88 units is reached, whichever occurs first.	Inactive
2021-05-17	13:21:19	Alexa	Take 43 units of Basaglar every morning.	If average fasting blood sugar is between 131 and 139, the insulin dose will be increased by 1 unit(s) every 3 day(s). If average fasting blood sugar is between 140 and 180, the insulin dose will be increased by 10 percent every 3 day(s). If average fasting blood sugar is greater than or equal to 181, the insulin dose will be increased by 20 percent every 3 day(s). Titration will stop when blood sugars are within goal, or when a max dose of 88 units is reached, whichever occurs first.	Inactive
2021-05-14	16:00:44	Alexa	Take 42 units of Basaglar every morning.	If average fasting blood sugar is between 131 and 139, the insulin dose will be increased by 1 unit(s) every 3 day(s). If average fasting blood sugar is between 140 and 180, the insulin dose will be increased by 10 percent every 3 day(s). If average fasting blood sugar is greater than or equal to 181, the insulin dose will be increased by 20 percent every 3 day(s). Titration will stop when blood sugars are within goal, or when a max dose of 88 units is reached, whichever occurs first.	Inactive

Providers can view insulin titration history on our custom web portal. Titration history listed the date and time of each new prescription, whether the prescription was changed by a provider via the portal or by the VBAI (Alexa), the insulin dose and frequency, and a summary of the titration protocol.

© 2023 Nayak A et al. *JAMA Network Open.*

eFigure 3. Physician Portal: Viewing Blood Glucose Logs

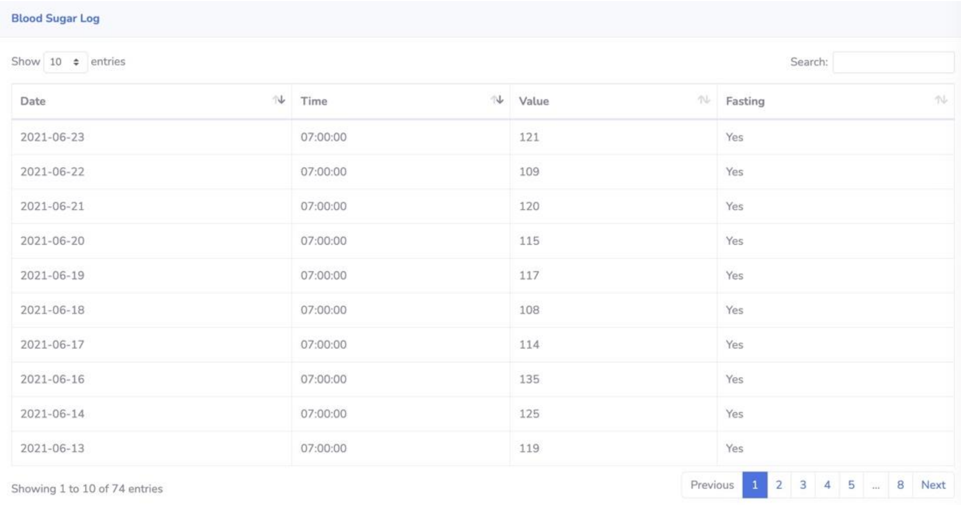

Blood Sugar Log

Show 10 entries   Search:

Date	Time	Value	Fasting
2021-06-23	07:00:00	121	Yes
2021-06-22	07:00:00	109	Yes
2021-06-21	07:00:00	120	Yes
2021-06-20	07:00:00	115	Yes
2021-06-19	07:00:00	117	Yes
2021-06-18	07:00:00	108	Yes
2021-06-17	07:00:00	114	Yes
2021-06-16	07:00:00	135	Yes
2021-06-14	07:00:00	125	Yes
2021-06-13	07:00:00	119	Yes

Showing 1 to 10 of 74 entries   Previous 1 2 3 4 5 … 8 Next

Blood sugar values logged with our voice AI were available for review by providers in real-time on our custom web portal.

© 2023 Nayak A et al. *JAMA Network Open.*

eFigure 4. Physician Portal: Viewing Insulin Logs

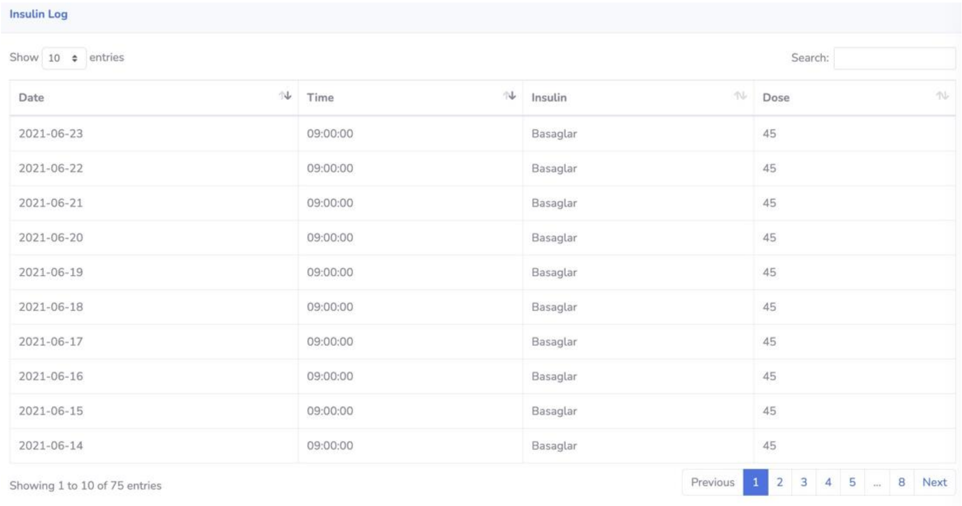

Insulin Log

Show 10 entries Search:

Date	Time	Insulin	Dose
2021-06-23	09:00:00	Basaglar	45
2021-06-22	09:00:00	Basaglar	45
2021-06-21	09:00:00	Basaglar	45
2021-06-20	09:00:00	Basaglar	45
2021-06-19	09:00:00	Basaglar	45
2021-06-18	09:00:00	Basaglar	45
2021-06-17	09:00:00	Basaglar	45
2021-06-16	09:00:00	Basaglar	45
2021-06-15	09:00:00	Basaglar	45
2021-06-14	09:00:00	Basaglar	45

Showing 1 to 10 of 75 entries

Previous 1 2 3 4 5 … 8 Next

Insulin use logged with our voice AI was available for review by providers in real-time on our custom web portal.

© 2023 Nayak A et al. *JAMA Network Open.*

eAppendix 4. Standard of Care Group Blood Glucose and Insulin Log

---

Start of Block: Blood Glucose and Insulin Log

This survey will be used to log your daily morning fasting blood sugar and your daily insulin dose. Please save your results and return to this survey throughout the course of your study to log your data. Submit your data once the log is complete.

Please input the first day of your trial. This date should be selected with your study coordinator: _________

Please log your blood sugars and insulin dose below. You can return to this log at any time. **Remember: a fasting sugar means you did not eat anything in the 8 hours before checking it.**

WEEK 1

STUDY DAY	DAY 1	DAY 2	DAY 3	DAY 4	DAY 5	DAY 6	DAY 7
**DATE** (Auto-populated based on start date above)							
**Fasting AM Sugar**	_____	_____	_____	_____	_____	_____	_____
**Insulin Dose**	_____	_____	_____	_____	_____	_____	_____

WEEK 2

STUDY DAY	DAY 8	DAY 9	DAY 10	DAY 11	DAY 12	DAY 13	DAY 14
**DATE**							
**Fasting AM Sugar**	_____	_____	_____	_____	_____	_____	_____
**Insulin Dose**	_____	_____	_____	_____	_____	_____	_____

WEEK 3

STUDY DAY	DAY 15	DAY 16	DAY 17	DAY 18	DAY 19	DAY 20	DAY 21
**DATE**							
**Fasting AM Sugar**	_____	_____	_____	_____	_____	_____	_____
**Insulin Dose**	_____	_____	_____	_____	_____	_____	_____

WEEK 4

STUDY DAY	DAY 22	DAY 23	DAY 24	DAY 25	DAY 26	DAY 27	DAY 28
**DATE**							
**Fasting AM Sugar**	_____	_____	_____	_____	_____	_____	_____
**Insulin Dose**	_____	_____	_____	_____	_____	_____	_____

WEEK 5

STUDY DAY	DAY 29	DAY 30	DAY 31	DAY 32	DAY 33	DAY 34	DAY 35
**DATE**							
**Fasting AM Sugar**	_____	_____	_____	_____	_____	_____	_____
**Insulin Dose**	_____	_____	_____	_____	_____	_____	_____

© 2023 Nayak A et al. *JAMA Network Open.*

WEEK 6

STUDY DAY	DAY 36	DAY 37	DAY 38	DAY 39	DAY 40	DAY 41	DAY 42
DATE							
Fasting AM Sugar	_____	_____	_____	_____	_____	_____	_____
Insulin Dose	_____	_____	_____	_____	_____	_____	_____

WEEK 7

STUDY DAY	DAY 43	DAY 44	DAY 45	DAY 46	DAY 47	DAY 48	DAY 49
DATE							
Fasting AM Sugar	_____	_____	_____	_____	_____	_____	_____
Insulin Dose	_____	_____	_____	_____	_____	_____	_____

WEEK 8

STUDY DAY	DAY 50	DAY 51	DAY 52	DAY 53	DAY 54	DAY 55	DAY 56
DATE							
Fasting AM Sugar	_____	_____	_____	_____	_____	_____	_____
Insulin Dose	_____	_____	_____	_____	_____	_____	_____

© 2023 Nayak A et al. *JAMA Network Open.*
