# Supplementary material for: Use of Voice-Based Conversational Artificial Intelligence for Basal Insulin Prescription Management Among Patients With Type 2 Diabetes: A Randomized Clinical Trial
Source: JAMA Netw Open. 2023 Dec 1;6(12):e2340232. doi: 10.1001/jamanetworkopen.2023.40232 (PMC10692866; doi:10.1001/jamanetworkopen.2023.40232)
Supplement: Supplement 3. — Data Sharing Statement [file jamanetwopen-e2340232-s003.pdf]

Data Sharing Statement

Nayak. Use of Voice-Based Conversational Artificial Intelligence for Basal Insulin Prescription Management Among Patients With Type 2 Diabetes. *JAMA Netw Open*. Published November 01, 2023. doi:10.1001/jamanetworkopen.2023.40232

Data
Data available: Yes
Data types: Deidentified participant data
How to access data: Requests for data can be sent to aknayak@stanford.edu
When available: With publication

Supporting Documents
Document types: Statistical/analytic code, Informed consent form
How to access documents: aknayak@stanford.edu
When available: With publication

Additional Information
Who can access the data: Anyone requesting the data
Types of analyses: For any purpose
Mechanisms of data availability: No special agreement needed
